# Supplementary material for: Recorded poor insight as a predictor of service use outcomes: cohort study of patients with first-episode psychosis in a large mental healthcare database
Source: BMJ Open. 2019 Jun 12;9(6):e028929. doi: 10.1136/bmjopen-2019-028929 (PMC6577359; doi:10.1136/bmjopen-2019-028929)
Supplement: Supplementary Table 1 [file bmjopen-2019-028929supp001.pdf]

Supplementary Table 1: Recorded insight and clinical outcomes at 12, 24, 36, 48 and 60 months

	Recorded poor insight	
	Present	Absent
	(n=991)	*(n=1,035)*
**12 months**		
Psychiatric hospitalisation (%)	33.76	18.84
Compulsory hospitalisation (%)	31.07	12.37
Number of unique antipsychotics (mean, SD)	1.86 (1.17)	1.34 (1.11)
Number of days spent as an inpatient (mean, SD)	38.92 (61.85)	12.49 (36.05)
**24 months**	*(n=762)*	*(n=976)*
Psychiatric hospitalisation (%)	44.88	28.48
Compulsory hospitalisation (%)	39.50	20.29
Number of unique antipsychotics (mean, SD)	1.92 (1.44)	1.90 (1.44)
Number of days spent as an inpatient (mean, SD)	42.91 (89.96)	40.87 (88.93)
**36 months**	*(n=607)*	*(n=854)*
Psychiatric hospitalisation (%)	49.26	33.96
Compulsory hospitalisation (%)	43.99	25.18
Number of unique antipsychotics (mean, SD)	2.14 (1.63)	2.06 (1.62)
Number of days spent as an inpatient (mean, SD)	60.97 (125.71)	54.68 (118.76)
**48 months**	*(n=462)*	*(n=723)*
Psychiatric hospitalisation (%)	53.68	38.45
Compulsory hospitalisation (%)	47.40	28.63
Number of unique antipsychotics (mean, SD)	2.34 (1.74)	2.16 (1.74)

Number of days spent as an inpatient (mean, SD)	77.75 (160.97)	65.14 (146.18)
**60 months**	*(n=331)*	*(n=595)*
Psychiatric hospitalisation (%)	56.19	41.51
Compulsory hospitalisation (%)	48.94	31.76
Number of unique antipsychotics (mean, SD)	2.48 (1.83)	2.24 (1.85)
Number of days spent as an inpatient (mean, SD)	92.44 (196.73)	74.01 (174.94)
