# Supplementary material for: Recorded poor insight as a predictor of service use outcomes: cohort study of patients with first-episode psychosis in a large mental healthcare database
Source: BMJ Open. 2019 Jun 12;9(6):e028929. doi: 10.1136/bmjopen-2019-028929 (PMC6577359; doi:10.1136/bmjopen-2019-028929)
Supplement: Author's manuscript [file bmjopen-2019-028929.draft_revisions.pdf]

BMJ Open is committed to open peer review. As part of this commitment we make the peer review history of every article we publish publicly available.

When an article is published we post the peer reviewers' comments and the authors' responses online. We also post the versions of the paper that were used during peer review. These are the versions that the peer review comments apply to.

The versions of the paper that follow are the versions that were submitted during the peer review process. They are not the versions of record or the final published versions. They should not be cited or distributed as the published version of this manuscript.

BMJ Open is an open access journal and the full, final, typeset and author-corrected version of record of the manuscript is available on our site with no access controls, subscription charges or pay-per-view fees (http://bmjopen.bmj.com).

If you have any questions on BMJ Open's open peer review process please email info.bmjopen@bmj.com

BMJ Open

Recorded poor insight as a predictor of service use outcomes in a cohort with first episode psychosis

Journal:	*BMJ Open*
Manuscript ID	bmjopen-2019-028929
Article Type:	Research
Date Submitted by the Author:	03-Jan-2019

Complete List of Authors:	Ramu, Neha<br>Kolliakou, Anna; King's College London, Psychological Medicine<br>Jyoti, Jyoti<br>Patel, Rashmi<br>Stewart, Robert; King's College London, Institute of Psychiatry

Keywords:	insight, psychosis, mental health outcomes, service use outcomes, natural language processing, CRIS

SCHOLARONE™
Manuscripts

Recorded poor insight as a predictor of service use outcomes in a cohort with first episode psychosis

Neha Ramu [1], Anna Kolliakou [1], Jyoti Jyoti [2], Rashmi Patel [1,2], Robert Stewart [1,2]

[1] Institute of Psychiatry, Psychology and Neuroscience, King's College London, London, United Kingdom

[2] South London and Maudsley NHS Foundation Trust, London, United Kingdom

Corresponding Author: Anna Kolliakou, Biomedical Research Centre Nucleus, PO92, Institute of Psychiatry, Psychology and Neuroscience, De Crespigny Park, London SE5 8AF, UK (anna.kolliakou@kcl.ac.uk)

Keywords: insight, psychosis, mental health outcomes, service use outcomes, natural language processing, CRIS

Word count: 2,675

For peer review only

Abstract

Objectives: To investigate recorded poor insight in relation to mental health and service use outcomes in a cohort with first episode psychosis

Design: We developed a natural language processing algorithm to ascertain statements of poor or diminished insight.

Setting: The clinical record text at the South London and Maudsley NHS Trust in the UK was used.

Participants: We applied the algorithm to characterise a cohort of 2026 patients with first episode psychosis attending an early intervention service.

Primary and secondary outcome measures: Recorded poor insight within one month of registration was investigated in relation to i) incidence of psychiatric hospitalisation, ii) odds of legally-enforced hospitalisation, iii) number of days spent as a mental health inpatient and iv) number of different antipsychotic agents prescribed; outcomes were measured over varying follow-up periods from 12 months to 60 months, adjusting for a range of socio-demographic and clinical covariates.

Results: Recorded poor insight, present in 48.9% of the sample, was positively associated with youngest and oldest age groups, unemployment, and schizophrenia (compared to bipolar disorder), and was negatively associated with Asian ethnicity, married status, home ownership and recorded cannabis use. It was significantly associated with higher levels of all four outcomes over the succeeding 12 months. Associations with hospitalisation incidence and number of antipsychotics remained independently significant when measured over 60 and 48 months, respectively.

Conclusions: Recorded poor insight in people with recent onset psychosis predicted higher subsequent inpatient mental healthcare use. Improving insight might benefit patients' course of illness as well as reduce mental health service use.

For peer review only - http://bmjopen.bmj.com/site/about/guidelines.xhtml

Article Summary

Strengths and limitations

- Our study shows that clinician-recorded insight can be successfully extracted from an anonymised mental health record database
- We demonstrated the potential of natural language processing applied to routine healthcare records to derive novel information of clinical relevance
- Follow-up assessments were only feasible for those cases remaining in the geographic catchment area served by the Trust
- Causal pathways between insight and clinical outcomes remain to be explored

For peer review only

For peer review only - http://bmjopen.bmj.com/site/about/guidelines.xhtml

Introduction

Schizophrenia and other psychotic disorders have potentially severe impacts both on individuals and society, although their course and prognosis are variable. Insight, the awareness and appreciation by an individual of their psychopathology, has long been considered a determinant of outcome. Patients with poor insight are less likely to understand their illness; hence have been found to be less likely to adhere to treatment[1–3] and/or require more extensive treatment[4]. Many studies have concluded that poor insight is associated with stigma and worse social performance; however, some have claimed that insight is not in fact directly linked to the outcome of the illness, but how it is progressing[5]. Self-reported quality of life has been found to be higher in association with poor insight; this has been suggested as secondary to delusional beliefs[6,7], although good insight has been found to be associated with higher risk of depression in people with schizophrenia[8] and with suicidality[9,10]. On the other hand poor insight in schizophrenia has been associated with higher anxiety[11], with obsessive/compulsive symptoms[12], and with violent behaviour in some[13] but not in all[14] studies. In mania, poor insight has been associated with elation rather than irritability or psychosis[15]. However, others have concluded that there are no associations of insight either with symptoms or progression of schizophrenia[16].

Despite the range of studies exploring insight in psychotic disorders, we could find no direct investigations of associations with service use outcomes. In a large mental healthcare data resource, we therefore sought to develop a means of extracting descriptions of insight from the text fields of clinical records, and investigated whether recorded poor insight early after a first clinical presentation with psychosis predicted increased subsequent service use.

For peer review only

For peer review only - http://bmjopen.bmj.com/site/about/guidelines.xhtml

Methods

Setting and Data Sources

The data used in this study were obtained from the South London and Maudsley NHS Foundation Trust (SLaM), one of Europe's largest mental healthcare organisations which provides comprehensive services across all ages and specialties to a defined geographic catchment of around 1.2 million residents within four south London boroughs (Lambeth, Southwark, Lewisham and Croydon). SLaM has used fully electronic health records for over ten years and its Clinical Record Interactive Search (CRIS) tool, set up in 2008[17,18], allows researcher access to de-identified data from the full record within a robust governance framework[19].

Exposure of interest and data extraction

CRIS has been substantially enhanced through natural language processing algorithms applied to extract constructs of interest from text fields in the source record using information extraction / named entity recognition techniques[18,20]. For this study, Text Hunter annotation software[21] was used to create training and test corpora classifying mentions of insight in the clinical record in order to train a supervised machine learning algorithm to recognise this automatically across the wider sample. An initial keyword search was carried out to extract sentences containing the word "insight" and a human annotator manually categorized these as either 'good insight' (for example, when insight was described as "clear", "improving", "partial", "good", "insightful", "present", "intact" and "aware"), 'poor insight' (e.g. described as "lacking", "poor", "limited", "insightless", "absent", "impaired", "lost" or words to that effect), or as not relevant (i.e. unclear/lengthy descriptions, unassessed insight, insight mentioned as a future goal rather that at the present, or where the level of insight was not immediately obvious). For generating training and independent test sets the algorithm, a randomly selected 1814 relevant sentences were manually annotated from all patients on CRIS with a previous diagnosis of schizophreniform or affective disorder (ICD-10 F2x or F3x), of which 788 were classified as having good insight, 826 as having poor insight and 200 as non-relevant statements. The algorithm generated classified 'poor insight' instances with 0.73 precision (positive predictive value) and 0.83 recall (sensitivity) against the manual gold standard.

For peer review only - http://bmjopen.bmj.com/site/about/guidelines.xhtml

Participants

For the analysis, a database was used which had been previously prepared via CRIS for an analysis of psychosis outcomes associated with cannabis use[22]. In summary, this comprised all 2026 individuals with first episode psychosis who were accepted by a SLaM early intervention service between 1st April 2006 and 31st March 2013. Outcome data were collected up to 31 March 2014. Predictor, covariate and outcome variable data were obtained via CRIS. Besides insight, the following covariates were ascertained using values recorded closest to the date of being accepted by an early intervention service: age, gender, ethnicity, marital status, employment status, type of accommodation, primary diagnosis and cannabis use. Ethnicity was recorded according to categories defined by the UK Office for National Statistics and was condensed for this analysis into four groups (White, Black, Asian, other). Diagnosis was recorded using the 10th edition of the International Classification of Diseases (ICD-10) classification system. The derivation of cannabis use through natural language processing and its application as a covariate have been previously described[22]. Using the natural language processing algorithm described above, recorded poor insight was ascertained from case records within one month either side of the date each patient was accepted to the early intervention service and this was defined as the primary exposure.

Outcomes

We investigated the association between poor insight and the following mental healthcare outcomes: i) number of psychiatric hospital admission, ii) any legally enforced (compulsory) admission under the UK Mental Health Act (MHA) iii) the number of unique antipsychotics prescribed (as a proxy measure of treatment failure) and iv) number of days spent in psychiatric hospital over a given follow-up period. The MHA is a UK statute law which allows compulsory admission for up to 28 days ('Section 2') or up to 6 months ('Section 3'). Antipsychotics used were ascertained both from structured fields and a natural language processing algorithm[18].

Statistical analysis

All participants were assessed for outcomes within 12 months of the date of being accepted to an early intervention service. Those with sufficient follow-up data were then also assessed for outcomes within 24, 36, 48 and 60 months of this first acceptance date (i.e. different but overlapping follow-up periods). This was an identical approach to that previous adopted for

For peer review only - http://bmjopen.bmj.com/site/about/guidelines.xhtml

analyses in these data[22], investigating discrete periods of follow-up time rather than using survival analysis because of the non-proportionality of hazards. The sample was first described and factors associated with poor insight investigated. Regression models were then used to evaluate unadjusted and successively adjusted associations with the four outcomes over the five different follow-up periods. Owing to over-dispersion, previously described for these data[22], we aimed to assess associations with number of hospital admissions and number of unique antipsychotic medications using multivariable negative binomial regression (zero-inflation having been investigated but giving rise to no meaningful difference). However, one of the models failed to converge and so Poisson regressions were used instead. Associations with legally enforced hospitalisation were assessed using multivariable binary logistic regression. Associations with number of inpatient days within given observation periods were investigated using multiple linear regression models. Reference groups for covariates were defined as those with the highest prevalence for each variable, and missing categories were included as predictor variables, so that no patients were excluded because of missing covariate data. Stata software version 13 (Statacorp Stata Statistical Software: Release 13; College Station, TX: StataCorp LP, 2011) was used.

Patient and public involvement

We did not directly incorporate PPI into this particular analysis but the SLaM BRC Case Register used in the study was developed with extensive PPI and is overseen by committees that include service-user and general public representatives.

For peer review only

Results

Patients

From the cohort of 2,026 individuals, 991 (48.9%) had at least one recording of poor insight within one month either side of their registration with the early intervention service. The sample characteristics and their associations with recorded poor insight are summarised in Table 1. This was more common in the youngest and oldest members of the cohort, in those who were recorded as being a student or unemployed, and in those not recorded as using cannabis. It was least common in patients of Asian ethnicity, in those who were married, and in home owners. Poor insight was most commonly recorded in schizoaffective disorder, schizophrenia and 'other' diagnosis, and least common in bipolar disorder.

Unadjusted and adjusted main outcomes

Associations with service use outcomes in unadjusted and multivariable analyses are described in Tables 2-5. Adjusted associations of recorded poor insight with higher numbers of hospitalisation episodes (Table 2) were strongest when evaluated within 12 or 24 months of first referral, fell below statistical significance for 36- and 48-month end points but strengthened again for the 60-month estimation. Higher odds of legally enforced hospitalisations were most strongly associated with poor insight when evaluated within the first 12 months (Table 3), although remained raised at borderline significance for a number of the other time periods. Higher number of unique antipsychotics prescribed was also most strongly predicted by recorded poor insight when measured within the first 36 months, although associations persisted at statistical significance over 48 months (Table 4). Higher number of inpatient days, however, was only significantly associated with poor insight when measured within the first 12 months (Table 5).

For peer review only

Discussion

In a large cohort of cases with first episode psychosis drawn from a mental healthcare database, we developed an algorithm to detect recorded poor insight and investigated this as a predictor of four subsequent service use outcomes. Poor insight was, in summary, significantly and independently associated with higher number of hospitalisation episodes overall, higher odds of legally enforced hospitalisation, higher numbers of days spent as an inpatient, and higher numbers of unique antipsychotic agents prescribed. Associations with these outcomes were strongest when evaluated over the first 12 months of mental health service contact.

Loss of insight has long been considered a potentially important feature of psychotic disorders, and clearly establishing a therapeutic alliance is more challenging when insight is poor, accounting for associations found with reduced treatment adherence[3,4]. On the other hand, reduced awareness of a mental disorder has been suggested to a reduced personal impact of that disorder, accounting for associations found with better self-rated quality of life[6,7] and lower risk of depression and suicidality[8–10]. It is therefore understandable that there has been some controversy over whether poor insight has prognostic relevance. Our study focused on a range of outcomes derived from mental healthcare records and, as described above, found these to be worse in people recorded as having poor insight early in the course of their care.

Several factors were associated with recorded insight. Better insight in people who used cannabis or had drug induced psychosis is potentially interesting, as it suggests that psychotic symptoms in these patients may be less enduring and time limited in association with substance use so that by the time they are assessed, they have insight into the likely link between illicit substances and psychotic disorder. Further research might helpfully investigate cannabis discontinuation in order to establish whether patients who continue using cannabis have less insight than those who do not. Better insight in bipolar disorder could reflect the episodic nature of the illness.

Strengths of the study include the large sample size and naturalistic nature of the cohort and follow-up. However, key limitations need to be borne in mind when interpreting the findings. Considering the measurement of insight, the performance of the NLP algorithm was judged to be satisfactory and clearly represents an important step forward in routine data collection

For peer review only - http://bmjopen.bmj.com/site/about/guidelines.xhtml

(as structured fields in case records invariably fail to record this construct, thus rendering it invisible in conventional healthcare databases); furthermore, sub-optimal measurement of insight would have obscured rather than exaggerated the prospective associations with the outcomes of interest. However, clearly statements about insight have to be recorded in the first place and there may be clinical circumstances and reasons which render these more or less likely. In addition, the construct cannot be assumed to be identical to an assessment of insight in a research interview, and we solely focused on recorded poor insight and did not seek to sub-characterise the sample into those with mixed good or poor statements. In terms of follow-up, hospitalisations and other outcomes would only be ascertained for those cases who remained in the geographic catchment served by SLaM, so out-migration might have affected longer-interval findings. In this analysis, as with a previous analysis of cannabis use as a risk factor in this sample[22] we investigated associations over different time periods. Longer follow-up evaluations clearly provide a more informed picture of prognosis; however, insight cannot be assumed to be constant over time and we did not attempt to quantify these trajectories – for example, more effective treatment may result in a virtuous cycle involving improved insight and better therapeutic engagement.

Residual confounding cannot be absolutely excluded, and causal pathways also remain to be elucidated; however, these might include failure to establish initial engagement with services resulting in symptomatic deterioration and requirement for inpatient care – particularly supported by the higher use of legally enforced hospitalisation. It is possible that poor insight at first presentation is associated with antipsychotic treatment failure, as suggested by the higher number of antipsychotics used, although it is difficult to draw this conclusion with certainty because of potentially complex interactions between insight and treatment effects. Poor insight might place strains on social support networks and compromise the role of protective factors, accounting for the observed associations between poor insight and indicators of social/financial disadvantage in our cohort. It might result in risk behaviours which result in worse outcomes, although we adjusted for cannabis use as one of these potential pathways and this did not account substantially for the associations observed. Finally, it is possible that poor insight is not a risk factor itself, but is a marker of a disorder which is already more severe in other respects (such as symptomatically or in terms of functional deterioration).

For peer review only - http://bmjopen.bmj.com/site/about/guidelines.xhtml

Conclusion

Our findings do support an important prognostic role for poor insight in people with psychotic disorders when this is mentioned early after first clinical presentation. Although economic modelling was not attempted, clearly outcomes such as number and duration of hospitalisation episodes have substantial impact, and measures taken to improve insight might similarly bring important benefits at a service level as well as on individuals' course of illness.

For peer review only

For peer review only - http://bmjopen.bmj.com/site/about/guidelines.xhtml

Patient consent for publication: Not required.

Author contributions: The study was conceived by NR, RS and RP. Natural language processing applications were developed by NR, AK and JJ. Analyses were carried out by NR and AK. NR led the preparation of the final report, to which all authors contributed significantly, approving the final version.

Funding: RS, RP, JJ and AK are part-funded by the National Institute for Health Research (NIHR) Biomedical Research Centre and Dementia Biomedical Research Unit at South London and Maudsley NHS Foundation Trust and King's College London.

Disclaimer: The funder had no role in study design; data collection, analysis, or interpretation; or writing of the article.

Competing interests: None declared

Ethics approval: Oxford C Research Ethics Committee, reference 08/H0606/71+5

Data sharing statement: There are no additional data available.

For peer review only

References

1. Niolu C, Barone Y, Bianciardi E, et al. Predictors of poor adherence to treatment in inpatients with bipolar and psychotic spectrum disorders. *Riv Psichiatr*. 2015;50(6):285–94. doi:10.1708/2098.22686

2. Lysaker P, Bell M, Milstein R, Bryson G, Beam-Goulet J. Insight and psychosocial treatment compliance in schizophrenia. *Psychiatry*. 1994;57(4):307–15.

3. Vohs JL, George S, Leonhardt BL, Lysaker PH. An integrative model of the impairments in insight in schizophrenia: emerging research on causal factors and treatments. *Expert Rev Neurother*. 2016;16(10):1193–204. doi:10.1080/14737175.2016.1199275

4. Bota RG, Munro JS, Ricci WF, Bota DA. The Dynamics of Insight in the Prodrome of Schizophrenia. *CNS Spectr*. 2006;11(5):355–62. doi:10.1017/S1092852900014486

5. Jacob K. Insight in psychosis: An indicator of severity of psychosis, an explanatory model of illness, and a coping strategy. *Indian J Psychol Med*. 2016;38(3):194–201. doi: 10.4103/0253-7176.183078

6. Hayhurst KP, Massie JA, Dunn G, Lewis SW, Drake RJ. Validity of subjective versus objective quality of life assessment in people with schizophrenia. *BMC Psychiatry*. 2014 Dec 24;14(1):365. doi: 10.1186/s12888-014-0365-x.

7. Roseman AS, Kasckow J, Fellows I, et al. Insight, quality of life, and functional capacity in middle-aged and older adults with schizophrenia. *Int J Geriatr Psychiatry*. 2008;23(7):760–5. doi: 10.1002/gps.1978

8. Drake RJ, Pickles A, Bentall RP, et al. The evolution of insight, paranoia and depression during early schizophrenia. *Psychol Med*. 2004;34(2):285–92. doi:10.1017/S0033291703008821

9. Kim CH, Jayathilake K, Meltzer HY. Hopelessness, neurocognitive function, and insight in schizophrenia: Relationship to suicidal behavior. *Schizophr Res*. 2003;60(1):71-80. doi: 10.1016/S0920-9964(02)00310-9

10. Amador XF, Friedman JH, Kasapis C, Yale SA, Flaum M, Gorman JM. Suicidal behavior in schizophrenia and its relationship to awareness of illness. *Am J Psychiatry*.

For peer review only

1996;153(9):1185–8. doi: 10.1176/ajp.153.9.1185

11. Stefanopoulou E, Lafuente AR, Saez Fonseca JA, Huxley A. Insight, global functioning and psychopathology amongst in-patient clients with schizophrenia. *Psychiatr Q*. 2009;80(3):155–65. doi:10.1007/s11126-009-9103-9

12. Catapano F, Sperandeo R, Perris F. Insight and Resistance in Patients with Obsessive-Compulsive Disorder. *Psychopathology*. 2001;34:62–8. doi:10.1159/000049282

13. Bonnet S, Lacambre M, Schandrin A, Capdevielle D, Courtet P. Insight and psychiatric dangerousness: A review of the literature. *Encephale*. 2017;43(2):146-153. doi: 10.1016/j.encep.2016.01.010

14. Köşger F, Eşsizoğlu A, Sönmez İ, Güleç G, Genek M, Akarsu Ö. The Relationship between Violence and Clinical Features, Insight and Cognitive Functions in Patients with Schizophrenia. *Turk Psikiyatri Derg*. 2016;27(2). Available from: http://www.turkpsikiyatri.com/en/default.aspx?modul=article&id=1028 (Accessed 9th November 2017)

15. Hanwella R, de Silva VA. Signs and symptoms of acute mania: A factor analysis. *BMC Psychiatry*. 2011;11:137. doi: 10.1186/1471-244X-11-137

16. Erickson MA, Lysaker PH. Self-esteem and insight as predictors of symptom change in schizophrenia: A longitudinal study. *Clin Schizophr Relat Psychoses*. 2012;6(2):69–75. doi: 10.3371/CSRP

17. Stewart R, Soremekun M, Perera G, et al. The South London and Maudsley NHS Foundation Trust Biomedical Research Centre (SLAM BRC) case register: development and descriptive data. *BMC Psychiatry*. 2009;9:51. doi: 10.1186/1471-244X-9-51

18. Perera G, Broadbent M, Callard F, Chang C. Cohort profile of the South London and Maudsley NHS Foundation Trust Biomedical Research Centre (SLaM BRC) Case Register: current status and recent. *BMJ open*. 2016;6:e008721. doi: 10.1136/bmjopen-2015-008721

19. Fernandes AC, Cloete D, Broadbent MT, et al. Development and evaluation of a de-identification procedure for a case register sourced from mental health electronic records. *BMC Medical Informatics and Decision Making*. 2013;13:71.

For peer review only - http://bmjopen.bmj.com/site/about/guidelines.xhtml

doi:10.1186/1472-6947-13-71

20. Jackson RG, Patel R, Jayatilleke N, et al. Natural language processing to extract symptoms of severe mental illness from clinical text: the Clinical Record Interactive Search Comprehensive Data Extraction (CRIS-CODE) project. *BMJ Open*. 2017;7(1):e012012. doi:10.1136/bmjopen-2016-012012

21. Jackson RG, Ball M, Patel R, Hayes RD, Dobson RJB, Stewart R. TextHunter--A User Friendly Tool for Extracting Generic Concepts from Free Text in Clinical Research. *AMIA Annual Symposium Proceedings*. 2014;2014:729–38. Available from: https://www.ncbi.nlm.nih.gov/pmc/articles/PMC4420012/ (Accessed November 9, 2017)

22. Patel R, Wilson R, Jackson R, et al. Association of cannabis use with hospital admission and antipsychotic treatment failure in first episode psychosis: an observational study. *BMJ Open*. 2016;6:e009888. doi: 10.1136/bmjopen-2015-009888

For peer review only

Table 1: Sample characteristics and associations with poor insight (n=2,026)

Variable	*Category*	*Number*	*% poor insight*	*Chi² (df) p-value*
Age	<16	19	73.68	9.64 (3)
	16-25	1,234	50.32	0.022
	26-35	747	45.65	
	>35	26	57.69	
Gender	Male	1,295	50.12	2.07 (1)
	Female	731	46.79	0.150
Ethnicity	White	616	49.19	8.22 (3)
	Asian	126	38.10	0.042
	Black	1,005	50.85	
	Other	279	46.24	
Relationship	Married	153	39.87	19.20 (3)
	Divorced	63	55.56	<0.001
	Single	1,727	48.52	
	Not recorded	83	68.67	
Employment	Employed	107	49.53	33.61 (3)
	Student	144	57.64	<0.001
	Unemployed	427	59.48	
	Not recorded	1,348	44.58	
Accommodation	Owner	14	28.57	97.78 (6)
	Private tenant	83	56.63	<0.001
	Council tenant	162	53.09	
	Supported	19	68.42	
	Homeless	37	51.35	
	Other	450	67.11	
	Not recorded	1,261	41.24	
Primary diagnosis	Schizophrenia	1,097	47.86	27.31 (5)
	Bipolar	100	30.00	<0.001

For peer review only

	Depression	94	44.68	
	Schizoaffective	35	54.29	
	Drug induced psychosis	63	39.68	
	Other	637	54.95	
History of cannabis use	No	1,087	58.97	94.90 (1)
	Yes	939	37.27	<0.001

For peer review only

Table 2: Association between poor insight and number of hospital admissions (negative binomial regression)

	Incidence rate ratio for the association with insight (95% confidence intervals, p-value)				
Time period evaluated	Unadjusted	Adj. age and gender	Adj. age, gender, ethnicity, relationship	Adj. age, gender, ethnicity, relationship, employment, accommodation	Adj. age, gender, ethnicity, relationship, employment, accommodation, diagnosis
12 months n=2026	1.37 (1.12, 1.67) 0.002	1.38 (1.13, 1.69) 0.001	1.36 (1.11, 1.67) 0.003	1.41 (1.14, 1.74) 0.001	1.37 (1.11, 1.70) 0.004
24 months n=1738	1.41 (1.15, 1.71) 0.001	1.42 (1.17, 1.74) <0.001	1.42 (1.17, 1.74) 0.001	1.46 (1.19, 1.80) <0.001	1.38 (1.12, 1.71) 0.003
36 months n=1461	1.26 (1.02, 1.55) 0.031	1.28 (1.03, 1.58) 0.024	1.29 (1.04, 1.60) 0.022	1.32 (1.06, 1.65) 0.015	1.22 (0.97, 1.54) 0.083
48 months n=1185	1.30 (1.03, 1.63) 0.029	1.32 (1.04, 1.66) 0.021	1.36 (1.07, 1.73) 0.013	1.36 (1.06, 1.74) 0.015	1.28 (1.00, 1.65) 0.053
60 months n=926	1.32 (1.01, 1.73) 0.042	1.34 (1.02, 1.76) 0.035	1.47 (1.10, 1.95) 0.008	1.46 (109, 1.95) 0.011	1.37 (1.02, 1.85) 0.036

For peer review only - http://bmjopen.bmj.com/site/about/guidelines.xhtml

Table 3: Association between insight and legally enforced hospitalisation* (logistic regression)

Odds ratio for the association with insight (95% confidence intervals, p-value)

Time period evaluated	Unadjusted	Adj. age and gender	Adj. age, gender, ethnicity, relationship	Adj. age, gender, ethnicity, relationship, employment, accommodation	Adj. age, gender, ethnicity, relationship, employment, accommodation, diagnosis
12 months n=2026	1.41 (1.13, 1.75) 0.002	1.41 (1.14, 1.76) 0.002	1.40 (1.12, 1.74) 0.003	1.42 (1.13, 1.78) 0.003	1.34 (1.07, 1.69) 0.012
24 months n=1738	1.35 (1.10, 1.67) 0.005	1.36 (1.10, 1.67) 0.004	1.36 (1.10, 1.68) 0.005	1.38 (1.10, 1.72) 0.004	1.29 (1.03, 1.62) 0.025
36 months n=1461	1.24 (0.99, 1.54) 0.058	1.25 (1.00, 1.55) 0.051	1.26 (1.01, 1.58) 0.042	1.29 (1.02, 1.62) 0.033	1.20 (0.95, 1.53) 0.125
48 months n=1185	1.26 (0.99, 1.61) 0.056	1.29 (1.01, 1.64) 0.041	1.33 (1.04, 1.71) 0.024	1.37 (1.06, 1.76) 0.017	1.30 (1.00, 1.68) 0.049
60 months n=926	1.12 (0.85, 1.47) 0.427	1.14 (0.86, 1.51) 0.356	1.24 (0.93, 1.66) 0.146	1.26 (0.93, 1.69) 0.136	1.19 (0.88, 1.62) 0.254

*Mental Health Act Section

For peer review only - http://bmjopen.bmj.com/site/about/guidelines.xhtml

Table 4: Association between insight and number of unique antipsychotics prescribed (poisson regression)

	Incidence rate ratio for the association with insight (95% confidence intervals, p-value)				
Time period evaluated	Unadjusted	Adj. age and gender	Adj. age, gender, ethnicity, relationship	Adj. age, gender, ethnicity, relationship, employment, accommodation	Adj. age, gender, ethnicity, relationship, employment, accommodation, diagnosis
12 months n=2026	1.27 (1.18, 1.36) <0.001	1.27 (1.18, 1.36) <0.001	1.25 (1.16, 1.34) <0.001	1.26 (1.17, 1.35) <0.001	1.24 (1.15, 1.33) <0.001
24 months n=1738	1.20 (1.12, 1.29) <0.001	1.20 (1.12, 1.29) <0.001	1.19 (1.11, 1.27) <0.001	1.20 (1.12, 1.29) <0.001	1.18 (1.10, 1.26) <0.001
36 months n=1461	1.15 (1.08, 1.24) <0.001	1.15 (1.07, 1.24) <0.001	1.15 (1.10, 1.23) <0.001	1.16 (1.08, 1.25) <0.001	1.13 (1.05, 1.22) <0.001
48 months n=1185	1.10 (1.02, 1.18) 0.016	1.10 (1.02, 1.19) 0.012	1.01 (1.02, 1.19) 0.013	1.11 (1.03, 1.20) 0.006	1.09 (1.01, 1.17) 0.034
60 months n=926	1.07 (0.98, 1.16) 0.133	1.07 (0.98, 1.16) 0.141	1.08 (0.99, 1.18) 0.062	1.09 (1.00, 1.19) 0.043	1.06 (0.98, 1.16) 0.153

For peer review only - http://bmjopen.bmj.com/site/about/guidelines.xhtml

Table 5: Association between insight and days spent hospitalised during the observation period (linear regression)

	B-coefficient for the association with recorded poor insight (95% confidence intervals, p-value)				
Time period evaluated	Unadjusted	Adj. age and gender	Adj. age, gender, ethnicity, relationship	Adj. age, gender, ethnicity, relationship, employment, accommodation	Adj. age, gender, ethnicity, relationship, employment, accommodation, diagnosis
12 months n=2026	9.57<br>(5.12, 14.0)<br><0.001	9.76<br>(5.30, 14.2)<br><0.001	9.46<br>(5.00, 13.9)<br><0.001	9.80<br>(5.24, 14.4)<br><0.001	9.12<br>(4.52, 13.7)<br><0.001
24 months n=1738	7.00<br>(-1.52, 15.5)<br>0.107	7.73<br>(-0.79, 16.3)<br>0.075	7.71<br>(-0.81, 16.2)<br>0.076	8.12<br>(-0.58, 16.8)<br>0.067)	6.56<br>(-2.20, 15.3)<br>0.142
36 months n=1461	4.54<br>(-8.15, 17.2)<br>0.483	6.06<br>(-6.61, 18.7)<br>0.348	6.70<br>(-5.93, 19.3)<br>0.298	6.57<br>(-6.26, 19.4)<br>0.316	4.17<br>(-8.75, 17.1)<br>0.527
48 months n=1185	-0.06<br>(-17.6, 17.5)<br>0.994	3.03<br>(-14.5, 20.6)<br>0.735	4.84<br>(-12.6, 23.0)<br>0.586	5.22<br>(-12.4, 22.8)<br>0.561	1.50<br>(-16.2, 19.2)<br>0.868
60 months n=926	-2.17<br>(-25.4, 21.0)<br>0.854	0.07<br>(-23.1, 23.3)<br>0.995	5.74<br>(-17.3, 28.8)<br>0.625	4.21<br>(-19.0, 27.4)<br>0.722	-1.01<br>(-24.3, 22.3)<br>0.932

For peer review only - http://bmjopen.bmj.com/site/about/guidelines.xhtml

Supplementary table 1: Recorded insight and clinical outcomes at 12, 24, 36, 48 and 60 months

	Recorded poor insight	
	Present (n=991)	Absent (n=1,035)
**12 months**		
Psychiatric hospitalisation (%)	22.6	28.6
Compulsory hospitalisation (%)	18.1	23.7
Number of unique antipsychotics (mean, SD)	1.39 (1.18)	1.77 (1.12)
Number of days spent as an inpatient (mean, SD)	19.7 (47.8)	29.3 (54.0)
**24 months**		
Psychiatric hospitalisation (%)	31.9	39.7
Compulsory hospitalisation (%)	25.7	31.2
Number of unique antipsychotics (mean, SD)	1.9 (1.45)	1.92 (1.44)
Number of days spent as an inpatient (mean, SD)	41.44 (89.1)	42.35 (89.8)
**36 months**		
Psychiatric hospitalisation (%)	37.8	43.3
Compulsory hospitalisation (%)	26.2	34.3
Number of unique antipsychotics (mean, SD)	2.07 (1.65)	2.13 (1.6)
Number of days spent as an inpatient (mean, SD)	56.7 (120.4)	58.9 (124.2)
**48 months**		
Psychiatric hospitalisation (%)	41.7	48.1
Compulsory hospitalisation (%)	33.7	39.1
Number of unique antipsychotics (mean, SD)	2.2 (1.78)	2.3 (1.70)
Number of days spent as an inpatient (mean, SD)	69.1 (148.7)	73.8 (158.5)
**60 months**		
Psychiatric hospitalisation (%)	44.2	51.2
Compulsory hospitalisation (%)	36.9	39.6
Number of unique antipsychotics (mean, SD)	2.3 (1.92)	2.4 (1.78)
Number of days spent as an inpatient (mean, SD)	80.37 (178.1)	86.1 (193.5)

For peer review only

STROBE Statement—checklist of items that should be included in reports of observational studies

	Item No	Recommendation
**Title and abstract**	1	(*a*) Indicate the study's design with a commonly used term in the title or the abstract<br>**PAGE 1**
		(*b*) Provide in the abstract an informative and balanced summary of what was done and what was found<br>**PAGE 2**
**Introduction**		
Background/rationale	2	Explain the scientific background and rationale for the investigation being reported<br>**PAGE 4**
Objectives	3	State specific objectives, including any pre-specified hypotheses<br>**PAGE 4**
**Methods**		
Study design	4	Present key elements of study design early in the paper<br>**PAGE 5**
Setting	5	Describe the setting, locations, and relevant dates, including periods of recruitment, exposure, follow-up, and data collection<br>**PAGES 5**
Participants	6	(*a*) *Cohort study*—Give the eligibility criteria, and the sources and methods of selection of participants. Describe methods of follow-up<br>*Case-control study*—Give the eligibility criteria, and the sources and methods of case ascertainment and control selection. Give the rationale for the choice of cases and controls<br>*Cross-sectional study*—Give the eligibility criteria, and the sources and methods of selection of participants
		(*b*) *Cohort study*—For matched studies, give matching criteria and number of exposed and unexposed<br>*Case-control study*—For matched studies, give matching criteria and the number of controls per case<br>**PAGE 6**
Variables	7	Clearly define all outcomes, exposures, predictors, potential confounders, and effect modifiers. Give diagnostic criteria, if applicable<br>**PAGES 6 and 7**
Data sources/ measurement	8*	For each variable of interest, give sources of data and details of methods of assessment (measurement). Describe comparability of assessment methods if there is more than one group<br>**PAGE 5**
Bias	9	Describe any efforts to address potential sources of bias<br>**PAGE 7**
Study size	10	Explain how the study size was arrived at<br>**PAGES 5 and 6**
Quantitative variables	11	Explain how quantitative variables were handled in the analyses. If applicable, describe which groupings were chosen and why<br>**PAGES 6 and7**
Statistical methods	12	(*a*) Describe all statistical methods, including those used to control for confounding<br>**PAGES 6 and 7**

For peer review only

For peer review only - http://bmjopen.bmj.com/site/about/guidelines.xhtml

		(*b*) Describe any methods used to examine subgroups and interactions<br>**PAGES 6 and 7**
		(*c*) Explain how missing data were addressed
		(*d*) *Cohort study*—If applicable, explain how loss to follow-up was addressed<br>*Case-control study*—If applicable, explain how matching of cases and controls was addressed<br>*Cross-sectional study*—If applicable, describe analytical methods taking account of sampling strategy
		(*e*) Describe any sensitivity analyses
**Results**		
Participants	13*	**PAGE 8**
Descriptive data	14*	**PAGE 8**
Outcome data	15*	**PAGE 8**
Main results	16	(*a*) Give unadjusted estimates and, if applicable, confounder-adjusted estimates and their precision (eg, 95% confidence interval). Make clear which confounders were adjusted for and why they were included<br>**PAGE 8**
		(*b*) Report category boundaries when continuous variables were categorized<br>**PAGE 8**
		(*c*) If relevant, consider translating estimates of relative risk into absolute risk for a meaningful time period
Other analyses	17	Report other analyses done—eg analyses of subgroups and interactions, and sensitivity analyses
**Discussion**		
Key results	18	Summarise key results with reference to study objectives<br>**PAGE 9**
Limitations	19	Discuss limitations of the study, taking into account sources of potential bias or imprecision. Discuss both direction and magnitude of any potential bias<br>**PAGES 9 and 10**
Interpretation	20	Give a cautious overall interpretation of results considering objectives, limitations, multiplicity of analyses, results from similar studies, and other relevant evidence<br>**PAGES 9 and 10**
Generalisability	21	Discuss the generalisability (external validity) of the study results<br>**PAGES 9 and 10**
**Other information**		
Funding	22	Give the source of funding and the role of the funders for the present study and, if applicable, for the original study on which the present article is based<br>**PAGE 12**

For peer review only

*Give information separately for cases and controls in case-control studies and, if applicable, for exposed and unexposed groups in cohort and cross-sectional studies.

Note: An Explanation and Elaboration article discusses each checklist item and gives methodological background and published examples of transparent reporting. The STROBE checklist is best used in conjunction with this article (freely available on the Web sites of PLoS Medicine at http://www.plosmedicine.org/, Annals of Internal Medicine at http://www.annals.org/, and Epidemiology at http://www.epidem.com/). Information on the STROBE Initiative is available at www.strobe-statement.org.

For peer review only

BMJ Open

Recorded poor insight as a predictor of service use outcomes: a cohort study of patients with first episode psychosis in a large mental healthcare database

Journal:	*BMJ Open*
Manuscript ID	bmjopen-2019-028929.R1
Article Type:	Research
Date Submitted by the Author:	18-Mar-2019
Complete List of Authors:	Ramu, Neha<br>Kolliakou, Anna; King's College London, Psychological Medicine<br>Jyoti, Jyoti<br>Patel, Rashmi<br>Stewart, Robert; King's College London, Institute of Psychiatry
**Primary Subject Heading**:	Mental health
Secondary Subject Heading:	Epidemiology, Health informatics
Keywords:	insight, psychosis, mental health outcomes, service use outcomes, natural language processing, CRIS

SCHOLARONE™
Manuscripts

Recorded poor insight as a predictor of service use outcomes: a cohort study of patients with first episode psychosis in a large mental healthcare database

Neha Ramu [1], Anna Kolliakou [1], Jyoti Jyoti [2], Rashmi Patel [1,2], Robert Stewart [1,2]

[1] Institute of Psychiatry, Psychology and Neuroscience, King's College London, London, United Kingdom

[2] South London and Maudsley NHS Foundation Trust, London, United Kingdom

Corresponding Author: Anna Kolliakou, Biomedical Research Centre Nucleus, PO92, Institute of Psychiatry, Psychology and Neuroscience, De Crespigny Park, London SE5 8AF, UK (anna.kolliakou@kcl.ac.uk)

Keywords: insight, psychosis, mental health outcomes, service use outcomes, natural language processing, CRIS

Word count: 2,960

For peer review only

For peer review only - http://bmjopen.bmj.com/site/about/guidelines.xhtml

Abstract

Objectives: To investigate recorded poor insight in relation to mental health and service use outcomes in a cohort with first episode psychosis

Design: We developed a natural language processing algorithm to ascertain statements of poor or diminished insight and tested this in a cohort of patients with first episode psychosis

Setting: The clinical record text at the South London and Maudsley NHS Trust in the UK was used.

Participants: We applied the algorithm to characterise a cohort of 2026 patients with first episode psychosis attending an early intervention service.

Primary and secondary outcome measures: Recorded poor insight within one month of registration was investigated in relation to i) incidence of psychiatric hospitalisation, ii) odds of legally-enforced hospitalisation, iii) number of days spent as a mental health inpatient and iv) number of different antipsychotic agents prescribed; outcomes were measured over varying follow-up periods from 12 months to 60 months, adjusting for a range of socio-demographic and clinical covariates.

Results: Recorded poor insight, present in 48.9% of the sample, was positively associated with youngest and oldest age groups, unemployment, and schizophrenia (compared to bipolar disorder), and was negatively associated with Asian ethnicity, married status, home ownership and recorded cannabis use. It was significantly associated with higher levels of all four outcomes over the succeeding 12 months. Associations with hospitalisation incidence and number of antipsychotics remained independently significant when measured over 60 and 48 months, respectively.

Conclusions: Recorded poor insight in people with recent onset psychosis predicted higher subsequent inpatient mental healthcare use. Improving insight might benefit patients' course of illness as well as reduce mental health service use.

For peer review only - http://bmjopen.bmj.com/site/about/guidelines.xhtml

Article Summary

Strengths and limitations

- Our study included a large sample size, followed a naturalistic method of cohort identification and follow-up and applied natural language processing, a novel text extraction method, to ascertain insight.
- Measurement of insight (as a binary fixed variable) depended on this clearly having been stated in the clinical record and cannot be assumed to be identical to assessment through interview
- Follow-up assessments were only feasible for those cases remaining in the geographic catchment area served by the Trust
- Causal pathways between insight and clinical outcomes cannot be determined by our analysis

For peer review only

For peer review only - http://bmjopen.bmj.com/site/about/guidelines.xhtml

Introduction

Schizophrenia and other psychotic disorders have potentially severe impacts both on individuals and society, although their course and prognosis are variable. The concept of insight has historically been challenging to define and measure. Currently, researchers and clinicians utilise long and short cognitive and clinical assessment schedules which measure uni- and multi-dimensional aspects of insight such as awareness of illness and its consequences, attribution of symptoms, acceptance of treatment and understanding of its effects[1,2] as well as cognitive notions such as self-reflection and self-centainty[3]. Other views additionally propose that insight not only depends on cognitive functioning but on a patient's cultural and life experiences that cannot accurately be measured through traditional objective assessments[4].

The awareness and appreciation by an individual of their psychopathology, has long been considered a determinant of outcome. Patients with poor insight are less likely to understand their illness; hence have been found to be less likely to adhere to treatment[5-7] and/or require more extensive treatment[8]. Many studies have concluded that poor insight is associated with stigma and worse social performance; however, some have claimed that insight is not in fact directly linked to the outcome of the illness, but how it is progressing[9]. Self-reported quality of life has been found to be higher in patients with poor insight; this has been suggested as secondary to delusional beliefs[10,11], although good insight has been found to be associated with higher risk of depression in people with schizophrenia[12] and with suicidality[13,14]. On the other hand poor insight in schizophrenia has been associated with higher anxiety[15], with obsessive/compulsive symptoms1[6], and with violent behaviour in some[17] but not in all[18] studies. In mania, poor insight has been associated with elation rather than irritability or psychosis[19]. However, others have concluded that there are no associations of insight either with symptoms or progression of schizophrenia[20].

Despite the range of studies exploring insight in psychotic disorders, we could find no direct investigations of associations with service use outcomes. In a large mental healthcare data resource, we therefore sought to develop a means of extracting descriptions of insight from the text fields of clinical records, and investigated whether recorded poor insight early after a first clinical presentation with psychosis predicted increased subsequent service use.

For peer review only - http://bmjopen.bmj.com/site/about/guidelines.xhtml

Methods

Setting and Data Sources

The data used in this study were obtained from the South London and Maudsley NHS Foundation Trust (SLaM), one of Europe's largest mental healthcare organisations which provides comprehensive services across all ages and specialties to a defined geographic catchment of around 1.2 million residents within four south London boroughs (Lambeth, Southwark, Lewisham and Croydon). SLaM has used fully electronic health records for over ten years and its Clinical Record Interactive Search (CRIS) tool, set up in 2008[21,22], allows researcher access to de-identified data from the full record within a robust governance framework[23].

Exposure of interest and data extraction

CRIS has been substantially enhanced through natural language processing algorithms applied to extract constructs of interest from text fields in the source record using information extraction / named entity recognition techniques[22,24]. For this study, Text Hunter annotation software[25] was used to create training and test corpora classifying mentions of insight in the clinical record in order to train a supervised machine learning algorithm to recognise this automatically across the wider sample. An initial keyword search was carried out to extract sentences containing the word "insight" and a human annotator manually categorized these as either 'good insight' (for example, when insight was described as "clear", "improving", "partial", "good", "insightful", "present", "intact" and "aware"), 'poor insight' (e.g. described as "lacking", "poor", "limited", "insightless", "absent", "impaired", "lost" or words to that effect), or as not relevant (i.e. unclear/lengthy descriptions, unassessed insight, insight mentioned as a future goal rather that at the present, or where the level of insight was not immediately obvious). For generating training and independent test sets the algorithm, a randomly selected 1814 relevant sentences were manually annotated from all patients on CRIS with a previous diagnosis of schizophreniform or affective disorder (ICD-10 F2x or F3x), of which 788 were classified as having good insight, 826 as having poor insight and 200 as non-relevant statements. Precision (Positive Predictive Value) and Recall (sensitivity) were used as performance metrics based on conventional practice in text extraction evaluation[26]. The algorithm generated classified 'poor insight' instances with 0.73 precision (positive predictive value) and 0.83 recall (sensitivity) against the manual gold standard.

For peer review only - http://bmjopen.bmj.com/site/about/guidelines.xhtml

Participants

For the analysis, a database was used which had been previously prepared via CRIS for an analysis of psychosis outcomes associated with cannabis use[27]. In summary, this comprised all 2026 individuals with first episode psychosis who were accepted by a SLaM early intervention (EI) service between 1st April 2006 and 31st March 2013. Criteria for accepting patients in SLaM EI services follow those outlined in the 'Standards for Early Intervention in Psychosis Services – 1st Edition'[28]. Outcome data were collected up to 31 March 2014. All participants were assessed for outcomes within 12 months of the date of being accepted to an early intervention service (2026 person-years). Participants with sufficient follow-up data were also assessed for outcomes within 24 months (n=1738; 3476 person-years), 36 months (n=1461; 4383 person-years), 48 months (n=1185; 4740 person-years) and 60 months (n=926; 4630 person-years). Predictor, covariate and outcome variable data were obtained via CRIS. Besides insight, the following covariates were ascertained using values recorded closest to the date of being accepted by an early intervention service: age, gender, ethnicity, marital status, employment status, and type of accommodation, primary diagnosis and cannabis use. Ethnicity was recorded according to categories defined by the UK Office for National Statistics and was condensed for this analysis into four groups (White, Black, Asian, other). Diagnosis was recorded using the 10th edition of the International Classification of Diseases (ICD-10) classification system. The derivation of cannabis use through natural language processing and its application as a covariate have been previously described[27]. Using the natural language processing algorithm described above, recorded poor insight was ascertained from case records within one month either side of the date each patient was accepted to the early intervention service and this was defined as the primary exposure.

Outcomes

We investigated the association between poor insight and the following mental healthcare outcomes: i) number of psychiatric hospital admission, ii) any legally enforced (compulsory) admission under the UK Mental Health Act (MHA) iii) the number of unique antipsychotics prescribed (as a proxy measure of treatment failure) and iv) number of days spent in psychiatric hospital over a given follow-up period. The MHA is a UK statute law which allows compulsory admission for up to 28 days ('Section 2') or up to 6 months ('Section 3'). Antipsychotics used were ascertained both from structured fields and a natural language processing algorithm[22].

For peer review only - http://bmjopen.bmj.com/site/about/guidelines.xhtml

Statistical analysis

All participants were assessed for outcomes within 12 months of the date of being accepted to an early intervention service. Those with sufficient follow-up data were then also assessed for outcomes within 24, 36, 48 and 60 months of this first acceptance date (i.e. different but overlapping follow-up periods). This was an identical approach to that previous adopted for analyses in these data[27], investigating discrete periods of follow-up time rather than using survival analysis because of the non-proportionality of hazards. The sample was first described and factors associated with poor insight investigated. Regression models were then used to evaluate unadjusted and successively adjusted associations with the four outcomes over the five different follow-up periods. Owing to over-dispersion, previously described for these data[27], we aimed to assess associations with number of hospital admissions and number of unique antipsychotic medications using multivariable negative binomial regression (zero-inflation having been investigated but giving rise to no meaningful difference). However, one of the models failed to converge and so Poisson regressions were used instead. Associations with legally enforced hospitalisation were assessed using multivariable binary logistic regression. Associations with number of inpatient days within given observation periods were investigated using multiple linear regression models. Reference groups for covariates were defined as those with the highest prevalence for each variable, and missing categories were included as predictor variables, so that no patients were excluded because of missing covariate data. Stata software version 13 (Statacorp Stata Statistical Software: Release 13; College Station, TX: StataCorp LP, 2011) was used.

Patient and public involvement

We did not directly incorporate PPI into this particular analysis but the SLaM BRC Case Register used in the study was developed with extensive PPI and is overseen by committees that include service-user and general public representatives.

For peer review only - http://bmjopen.bmj.com/site/about/guidelines.xhtml

Results

Patients

From the cohort of 2,026 individuals, 991 (48.9%) had at least one recording of poor insight within one month either side of their registration with the early intervention service. The sample characteristics and their associations with recorded poor insight are summarised in Table 1. This was more common in the youngest and oldest members of the cohort, in those who were recorded as being a student or unemployed, and in those not recorded as using cannabis. It was least common in patients of Asian ethnicity, in those who were married, and in home owners. Poor insight was most commonly recorded in schizoaffective disorder, schizophrenia and 'other' diagnosis, and least common in bipolar disorder.

Unadjusted and adjusted main outcomes

Associations with service use outcomes in unadjusted and multivariable analyses are described in Tables 2-5. Adjusted associations of recorded poor insight with higher numbers of hospitalisation episodes (Table 2) were strongest when evaluated within 12 or 24 months of first referral, fell below statistical significance for analyses of 36- and 48-month follow-up periods but strengthened again for the 60-month estimation, although coefficients did not vary substantially. Higher odds of legally enforced hospitalisations were most strongly associated with poor insight when evaluated within the first 12 months (Table 3), although remained raised at borderline significance for a number of the other time periods. Higher number of unique antipsychotics prescribed was also most strongly predicted by recorded poor insight when measured within the first 36 months, although associations persisted at statistical significance over 48 months (Table 4). Higher number of inpatient days, however, was only significantly associated with poor insight when measured within the first 12 months (Table 5). For proportions of patients (with present or absent poor insight) and each clinical outcome at 12, 24, 36, 48 and 60 months, please see Supplementary Table 1.

For peer review only - http://bmjopen.bmj.com/site/about/guidelines.xhtml

Discussion

In a large cohort of cases with first episode psychosis drawn from a mental healthcare database, we developed an algorithm to detect recorded poor insight and investigated this as a predictor of four subsequent service use outcomes. Rate of poor insight in our cohort (48.9%) was in the range of that reported by studies assessing it through routine data collection methods (~50%)[29,30,31,32]. Poor insight was, in summary, significantly and independently associated with higher number of hospitalisation episodes overall, higher odds of legally enforced hospitalisation, higher numbers of days spent as an inpatient, and higher numbers of unique antipsychotic agents prescribed. Associations with these outcomes were strongest when evaluated over the first 12 months of mental health service contact.

Loss of insight has long been considered a potentially important feature of psychotic disorders, and clearly establishing a therapeutic alliance is more challenging when insight is poor, accounting for associations found with reduced treatment adherence[7,8]. On the other hand, reduced awareness of a mental disorder has been suggested to a reduced personal impact of that disorder, accounting for associations found with better self-rated quality of life[10,11] and lower risk of depression and suicidality[12-14]. It is therefore understandable that there has been some controversy over whether poor insight has prognostic relevance. Our study focused on a range of outcomes derived from mental healthcare records and, as described above, found these to be worse in people recorded as having poor insight early in the course of their care.

Several factors were associated with recorded insight. Better insight in people who used cannabis or had drug induced psychosis is potentially interesting, as it suggests that psychotic symptoms in these patients may be less enduring and time limited in association with substance use so that by the time they are assessed, they have insight into the likely link between illicit substances and psychotic disorder. Further research might helpfully investigate cannabis discontinuation in order to establish whether patients who continue using cannabis have less insight than those who do not. Better insight in bipolar disorder could reflect the episodic nature of the illness.

Strengths of the study include the large sample size and naturalistic nature of the cohort and follow-up. It has also demonstrated the great potential for NLP applied to routine healthcare records in deriving novel information of clinical relevance. However, key limitations need to

For peer review only - http://bmjopen.bmj.com/site/about/guidelines.xhtml

be borne in mind when interpreting the findings. Considering the measurement of insight, the performance of the NLP algorithm was judged to be satisfactory and clearly represents an important step forward in routine data collection (structured fields in case records invariably fail to record this construct thus rendering it invisible in conventional healthcare databases); furthermore, sub-optimal measurement of insight would have obscured rather than exaggerated the prospective associations with the outcomes of interest. However, clearly statements about insight have to be recorded in the first place and there may be clinical circumstances and reasons which render these more or less likely. For example, clinicians may be biased to record insight when it is poor or noticeably absent but not when it is present. In addition, as we measured insight as a fixed binary variable, the construct cannot be assumed to be identical to an assessment of insight in a research interview, and we solely focused on recorded poor insight and did not seek to sub-characterise the sample into those with mixed good or poor statements. Additionally, the precision and recall rates still allow for a risk of false positive and false negative instances of poor insight and further work could be employed to improve the performance metrics. In terms of follow-up, hospitalisations and other outcomes would only be ascertained for those cases which remained in the geographic catchment served by SLaM, so out-migration might have affected longer-interval findings. In this analysis, as with a previous analysis of cannabis use as a risk factor in this sample[22] we investigated associations over different time periods. Longer follow-up evaluations clearly provide a more informed picture of prognosis; however, insight cannot be assumed to be constant over time and we did not attempt to quantify these trajectories – for example, more effective treatment may result in a virtuous cycle involving improved insight and better therapeutic engagement.

Residual confounding cannot be absolutely excluded, and causal pathways also remain to be elucidated; however, these might include failure to establish initial engagement with services resulting in symptomatic deterioration and requirement for inpatient care – particularly supported by the higher use of legally enforced hospitalisation. It is possible that poor insight at first presentation is associated with antipsychotic treatment failure, as suggested by the higher number of antipsychotics used, although it is difficult to draw this conclusion with certainty because of potentially complex interactions between insight and treatment effects. Poor insight might place strains on social support networks and compromise the role of protective factors, accounting for the observed associations between poor insight and indicators of social/financial disadvantage in our cohort. It might result in risk behaviours

For peer review only - http://bmjopen.bmj.com/site/about/guidelines.xhtml

which result in worse outcomes, although we adjusted for cannabis use as one of these potential pathways and this did not account substantially for the associations observed. Finally, it is possible that poor insight is not a risk factor itself, but is a marker of a disorder which is already more severe in other respects (such as symptomatically or in terms of functional deterioration). Importantly, this study focused on the relationship between insight recorded shortly after presentation and the outcomes of interest, and we did not seek to capture changes in insight over the follow-up periods; this would be a potentially useful further line of enquiry, although dependent on the extent to which fluctuations in insight are recorded in routine mental healthcare.

Conclusion

Our findings do support an important prognostic role for poor insight in people with psychotic disorders when this is mentioned early after first clinical presentation. Although economic modelling was not attempted, clearly outcomes such as number and duration of hospitalisation episodes have substantial impact, and measures taken to improve insight might similarly bring important benefits at a service level as well as on individuals' course of illness.

For peer review only

For peer review only - http://bmjopen.bmj.com/site/about/guidelines.xhtml

Patient consent for publication: Not required.

Author contributions: The study was conceived by NR, RS and RP. Natural language processing applications were developed by NR, AK and JJ. Analyses were carried out by NR and AK. NR and AK led the preparation of the final report, to which all authors contributed significantly, approving the final version.

Funding: RS, RP, JJ and AK are part-funded by the National Institute for Health Research (NIHR) Biomedical Research Centre and Dementia Biomedical Research Unit at South London and Maudsley NHS Foundation Trust and King's College London.

Disclaimer: The funder had no role in study design; data collection, analysis, or interpretation; or writing of the article.

Competing interests: None declared

Ethics approval: Oxford C Research Ethics Committee, reference 08/H0606/71+5

Data sharing statement: All data relevant to the study are included in the article or uploaded as supplementary information

For peer review only

For peer review only - http://bmjopen.bmj.com/site/about/guidelines.xhtml

References

1. Birchwood M, Smith J, Drury V et al. A self-report Insight Scale for psychosis: reliability, validity and sensitivity to change. Acta Psychiatr Scand 1994;89:62–7.
2. Tranulis C, Lepage M, Ashok M. Insight in first episode psychosis: who is measuring what? *Early Interv Psych*. 2008;2:34-41.
3. Beck AT, Baruch E, Balter JM, Steer RA, Warman DM. A new instrument for measuring insight: the Beck Cognitive Insight Scale. *Schizophr Res.* 2004;68:319–29.
4. Kirmayer L, Corin E. Inside knowledge: cultural constructions of insight in psychosis. In: Amador XF, David AS, eds. *Insight and Psychosis: Awareness of Illness in Schizophrenia and Related Disorders, 2nd edn*. New York: Oxford University Press, 2004; 197–230.
5. Niolu C, Barone Y, Bianciardi E, et al. Predictors of poor adherence to treatment in inpatients with bipolar and psychotic spectrum disorders. *Riv Psichiatr*. 2015;50(6):285–94. doi:10.1708/2098.22686
6. Lysaker P, Bell M, Milstein R, Bryson G, Beam-Goulet J. Insight and psychosocial treatment compliance in schizophrenia. *Psychiatry*. 1994;57(4):307–15.
7. Vohs JL, George S, Leonhardt BL, Lysaker PH. An integrative model of the impairments in insight in schizophrenia: emerging research on causal factors and treatments. *Expert Rev Neurother*. 2016;16(10):1193–204. doi:10.1080/14737175.2016.1199275
8. Bota RG, Munro JS, Ricci WF, Bota DA. The Dynamics of Insight in the Prodrome of Schizophrenia. *CNS Spectr*. 2006;11(5):355–62. doi:10.1017/S1092852900014486
9. Jacob K. Insight in psychosis: An indicator of severity of psychosis, an explanatory model of illness, and a coping strategy. *Indian J Psychol Med*. 2016;38(3):194–201. doi: 10.4103/0253-7176.183078
10. Hayhurst KP, Massie JA, Dunn G, Lewis SW, Drake RJ. Validity of subjective versus objective quality of life assessment in people with schizophrenia. *BMC Psychiatry*. 2014 Dec 24;14(1):365. doi: 10.1186/s12888-014-0365-x.
11. Roseman AS, Kasckow J, Fellows I, et al. Insight, quality of life, and functional capacity in middle-aged and older adults with schizophrenia. *Int J Geriatr Psychiatry*. 2008;23(7):760–5. doi: 10.1002/gps.1978
12. Drake RJ, Pickles A, Bentall RP, et al. The evolution of insight, paranoia and

For peer review only - http://bmjopen.bmj.com/site/about/guidelines.xhtml

depression during early schizophrenia. *Psychol Med*. 2004;34(2):285–92. doi:10.1017/S0033291703008821

13. Kim CH, Jayathilake K, Meltzer HY. Hopelessness, neurocognitive function, and insight in schizophrenia: Relationship to suicidal behavior. *Schizophr Res*. 2003;60(1):71-80. doi: 10.1016/S0920-9964(02)00310-9
14. Amador XF, Friedman JH, Kasapis C, Yale SA, Flaum M, Gorman JM. Suicidal behavior in schizophrenia and its relationship to awareness of illness. *Am J Psychiatry.* 1996;153(9):1185–8. doi: 10.1176/ajp.153.9.1185
15. Stefanopoulou E, Lafuente AR, Saez Fonseca JA, Huxley A. Insight, global functioning and psychopathology amongst in-patient clients with schizophrenia. *Psychiatr Q*. 2009;80(3):155–65. doi:10.1007/s11126-009-9103-9
16. Catapano F, Sperandeo R, Perris F. Insight and Resistance in Patients with Obsessive-Compulsive Disorder. *Psychopathology*. 2001;34:62–8. doi:10.1159/000049282
17. Bonnet S, Lacambre M, Schandrin A, Capdevielle D, Courtet P. Insight and psychiatric dangerousness: A review of the literature. *Encephale*. 2017;43(2):146-153. doi: 10.1016/j.encep.2016.01.010
18. Köşger F, Eşsizoğlu A, Sönmez İ, Güleç G, Genek M, Akarsu Ö. The Relationship between Violence and Clinical Features, Insight and Cognitive Functions in Patients with Schizophrenia. *Turk Psikiyatri Derg*. 2016;27(2). Available from: http://www.turkpsikiyatri.com/en/default.aspx?modul=article&id=1028 (Accessed 9th November 2017)
19. Hanwella R, de Silva VA. Signs and symptoms of acute mania: A factor analysis. *BMC Psychiatry*. 2011;11:137. doi: 10.1186/1471-244X-11-137
20. Erickson MA, Lysaker PH. Self-esteem and insight as predictors of symptom change in schizophrenia: A longitudinal study. *Clin Schizophr Relat Psychoses*. 2012;6(2):69–75. doi: 10.3371/CSRP
21. Stewart R, Soremekun M, Perera G, et al. The South London and Maudsley NHS Foundation Trust Biomedical Research Centre (SLAM BRC) case register: development and descriptive data. *BMC Psychiatry*. 2009;9:51. doi: 10.1186/1471-244X-9-51
22. Perera G, Broadbent M, Callard F, Chang C. Cohort profile of the South London and Maudsley NHS Foundation Trust Biomedical Research Centre (SLaM BRC) Case Register: current status and recent. *BMJ open*. 2016;6:e008721. doi: 10.1136/bmjopen-2015-008721

For peer review only - http://bmjopen.bmj.com/site/about/guidelines.xhtml

23. Fernandes AC, Cloete D, Broadbent MT, et al. Development and evaluation of a de-identification procedure for a case register sourced from mental health electronic records. *BMC Medical Informatics and Decision Making*. 2013;13:71. doi:10.1186/1472-6947-13-71
24. Jackson RG, Patel R, Jayatilleke N, et al. Natural language processing to extract symptoms of severe mental illness from clinical text: the Clinical Record Interactive Search Comprehensive Data Extraction (CRIS-CODE) project. *BMJ Open*. 2017;7(1):e012012. doi:10.1136/bmjopen-2016-012012
25. Jackson RG, Ball M, Patel R, Hayes RD, Dobson RJB, Stewart R. TextHunter--A User Friendly Tool for Extracting Generic Concepts from Free Text in Clinical Research. *AMIA Annual Symposium Proceedings*. 2014; 729–38. Available from: https://www.ncbi.nlm.nih.gov/pmc/articles/PMC4420012/ (Accessed November 9, 2017)
26. Derczynski L. Complementarity, F-score, and NLP Evaluation. *Language Resources and Evaluation Proceedings*.2016. Available from: http://www.derczynski.com/sheffield/papers/f1_compl_eval.pdf/ (Accessed 14th March 2019)
27. Patel R, Wilson R, Jackson R, et al. Association of cannabis use with hospital admission and antipsychotic treatment failure in first episode psychosis: an observational study. *BMJ Open*. 2016;6:e009888. doi: 10.1136/bmjopen-2015-009888
28. Early Intervention in Psychosis Network. Standards for Early Intervention in Psychosis Services – 1st Edition. Royal College of Psychiatrists 2018; Available from https://www.rcpsych.ac.uk/docs/default-source/improving-care/ccqi/quality-networks/early-intervention-in-psychosis-teams-(eipn)/epin-standards-first-edition.pdf?sfvrsn=fd9b4a0f_2/ (Accessed March 14th 2019)
29. Sevy S, Nathanson K,Visweswaraiah H, Amador X. The relationship between insight and symptoms in schizophrenia, *Compr Psychiatry*. 2004;45:16-9.
30. Dickerson FB, Boronow JJ, Ringel N, Parente F. Lack of insight among outpatients with schizophrenia, *Psychiatr Serv.* 1997;48;195-9.
31. Lysaker P, Bryson GJ, Bell M. Insight and work performance in schizophrenia, *J Nerv Ment Dis*. 2002;190:142-6.
32. Gharabawi GM, Lasser RA, Bossie, CA, Zhu Y, Amador X. Insight and its relationship to clinical outcomes in patients with schizophrenia or schizoaffective disorder receiving long-acting risperidone, *Int Clin Psychopharmacol*. 2006;21:233-4

For peer review only - http://bmjopen.bmj.com/site/about/guidelines.xhtml

Table 1: Sample characteristics and associations with poor insight (n=2,026)

Variable	*Category*	*Number*	*% poor insight*	*χ^2 (df) p-value*
Age	<16	19	73.68	9.64 (3)
	16-25	1,234	50.32	0.022
	26-35	747	45.65	
	>35	26	57.69	
Gender	Male	1,295	50.12	2.07 (1)
	Female	731	46.79	0.150
Ethnicity	White	616	49.19	8.22 (3)
	Asian	126	38.10	0.042
	Black	1,005	50.85	
	Other	279	46.24	
Relationship	Married	153	39.87	19.20 (3)
	Divorced	63	55.56	<0.001
	Single	1,727	48.52	
	Not recorded	83	68.67	
Employment	Employed	107	49.53	33.61 (3)
	Student	144	57.64	<0.001
	Unemployed	427	59.48	
	Not recorded	1,348	44.58	
Accommodation	Owner	14	28.57	97.78 (6)
	Private tenant	83	56.63	<0.001
	Council tenant	162	53.09	
	Supported	19	68.42	
	Homeless	37	51.35	
	Other	450	67.11	
	Not recorded	1,261	41.24	
Primary diagnosis	Schizophrenia	1,097	47.86	27.31 (5)
	Bipolar	100	30.00	<0.001
	Depression	94	44.68	
	Schizoaffective	35	54.29	
	Drug induced	63	39.68	

For peer review only - http://bmjopen.bmj.com/site/about/guidelines.xhtml

	psychosis			
	Other	637	54.95	
History of cannabis use	No	1,087	58.97	94.90 (1)
	Yes	939	37.27	<0.001

For peer review only

For peer review only - http://bmjopen.bmj.com/site/about/guidelines.xhtml

Table 2: Association between poor insight and number of hospital admissions (negative binomial regression)

Time period evaluated	*Incidence rate ratio for the association with insight (95% confidence intervals, p-value)*				
	Unadjusted	Adj. age and gender	Adj. age, gender, ethnicity, relationship	Adj. age, gender, ethnicity, relationship, employment, accommodation	Adj. age, gender, ethnicity, relationship, employment, accommodation, diagnosis
12 months n=2026	1.37 (1.12, 1.67) 0.002	1.38 (1.13, 1.69) 0.001	1.36 (1.11, 1.67) 0.003	1.41 (1.14, 1.74) 0.001	1.37 (1.11, 1.70) 0.004
24 months n=1738	1.41 (1.15, 1.71) 0.001	1.42 (1.17, 1.74) <0.001	1.42 (1.17, 1.74) 0.001	1.46 (1.19, 1.80) <0.001	1.38 (1.12, 1.71) 0.003
36 months n=1461	1.26 (1.02, 1.55) 0.031	1.28 (1.03, 1.58) 0.024	1.29 (1.04, 1.60) 0.022	1.32 (1.06, 1.65) 0.015	1.22 (0.97, 1.54) 0.083
48 months n=1185	1.30 (1.03, 1.63) 0.029	1.32 (1.04, 1.66) 0.021	1.36 (1.07, 1.73) 0.013	1.36 (1.06, 1.74) 0.015	1.28 (1.00, 1.65) 0.053
60 months n=926	1.32 (1.01, 1.73) 0.042	1.34 (1.02, 1.76) 0.035	1.47 (1.10, 1.95) 0.008	1.46 (109, 1.95) 0.011	1.37 (1.02, 1.85) 0.036

For peer review only - http://bmjopen.bmj.com/site/about/guidelines.xhtml

Table 3: Association between insight and legally enforced hospitalisation* (logistic regression)

Time period evaluated	*Odds ratio for the association with insight (95% confidence intervals, p-value)* Unadjusted	Adj. age and gender	Adj. age, gender, ethnicity, relationship	Adj. age, gender, ethnicity, relationship, employment, accommodation	Adj. age, gender, ethnicity, relationship, employment, accommodation, diagnosis
12 months n=2026	1.41 (1.13, 1.75) 0.002	1.41 (1.14, 1.76) 0.002	1.40 (1.12, 1.74) 0.003	1.42 (1.13, 1.78) 0.003	1.34 (1.07, 1.69) 0.012
24 months n=1738	1.35 (1.10, 1.67) 0.005	1.36 (1.10, 1.67) 0.004	1.36 (1.10, 1.68) 0.005	1.38 (1.10, 1.72) 0.004	1.29 (1.03, 1.62) 0.025
36 months n=1461	1.24 (0.99, 1.54) 0.058	1.25 (1.00, 1.55) 0.051	1.26 (1.01, 1.58) 0.042	1.29 (1.02, 1.62) 0.033	1.20 (0.95, 1.53) 0.125
48 months n=1185	1.26 (0.99, 1.61) 0.056	1.29 (1.01, 1.64) 0.041	1.33 (1.04, 1.71) 0.024	1.37 (1.06, 1.76) 0.017	1.30 (1.00, 1.68) 0.049
60 months n=926	1.12 (0.85, 1.47) 0.427	1.14 (0.86, 1.51) 0.356	1.24 (0.93, 1.66) 0.146	1.26 (0.93, 1.69) 0.136	1.19 (0.88, 1.62) 0.254

*Mental Health Act Section

For peer review only - http://bmjopen.bmj.com/site/about/guidelines.xhtml

Table 4: Association between insight and number of unique antipsychotics prescribed (poisson regression)

	Incidence rate ratio for the association with insight (95% confidence intervals, p-value)				
Time period evaluated	Unadjusted	Adj. age and gender	Adj. age, gender, ethnicity, relationship	Adj. age, gender, ethnicity, relationship, employment, accommodation	Adj. age, gender, ethnicity, relationship, employment, accommodation, diagnosis
12 months n=2026	1.27 (1.18, 1.36) <0.001	1.27 (1.18, 1.36) <0.001	1.25 (1.16, 1.34) <0.001	1.26 (1.17, 1.35) <0.001	1.24 (1.15, 1.33) <0.001
24 months n=1738	1.20 (1.12, 1.29) <0.001	1.20 (1.12, 1.29) <0.001	1.19 (1.11, 1.27) <0.001	1.20 (1.12, 1.29) <0.001	1.18 (1.10, 1.26) <0.001
36 months n=1461	1.15 (1.08, 1.24) <0.001	1.15 (1.07, 1.24) <0.001	1.15 (1.10, 1.23) <0.001	1.16 (1.08, 1.25) <0.001	1.13 (1.05, 1.22) <0.001
48 months n=1185	1.10 (1.02, 1.18) 0.016	1.10 (1.02, 1.19) 0.012	1.01 (1.02, 1.19) 0.013	1.11 (1.03, 1.20) 0.006	1.09 (1.01, 1.17) 0.034
60 months n=926	1.07 (0.98, 1.16) 0.133	1.07 (0.98, 1.16) 0.141	1.08 (0.99, 1.18) 0.062	1.09 (1.00, 1.19) 0.043	1.06 (0.98, 1.16) 0.153

For peer review only

For peer review only - http://bmjopen.bmj.com/site/about/guidelines.xhtml

Table 5: Association between insight and days spent hospitalised during the observation period (linear regression)

Time period evaluated	*B-coefficient for the association with recorded poor insight (95% confidence intervals, p-value)*				
	Unadjusted	Adj. age and gender	Adj. age, gender, ethnicity, relationship	Adj. age, gender, ethnicity, relationship, employment, accommodation	Adj. age, gender, ethnicity, relationship, employment, accommodation, diagnosis
12 months n=2026	9.57 (5.12, 14.0) <0.001	9.76 (5.30, 14.2) <0.001	9.46 (5.00, 13.9) <0.001	9.80 (5.24, 14.4) <0.001	9.12 (4.52, 13.7) <0.001
24 months n=1738	7.00 (-1.52, 15.5) 0.107	7.73 (-0.79, 16.3) 0.075	7.71 (-0.81, 16.2) 0.076	8.12 (-0.58, 16.8) 0.067)	6.56 (-2.20, 15.3) 0.142
36 months n=1461	4.54 (-8.15, 17.2) 0.483	6.06 (-6.61, 18.7) 0.348	6.70 (-5.93, 19.3) 0.298	6.57 (-6.26, 19.4) 0.316	4.17 (-8.75, 17.1) 0.527
48 months n=1185	-0.06 (-17.6, 17.5) 0.994	3.03 (-14.5, 20.6) 0.735	4.84 (-12.6, 23.0) 0.586	5.22 (-12.4, 22.8) 0.561	1.50 (-16.2, 19.2) 0.868
60 months n=926	-2.17 (-25.4, 21.0) 0.854	0.07 (-23.1, 23.3) 0.995	5.74 (-17.3, 28.8) 0.625	4.21 (-19.0, 27.4) 0.722	-1.01 (-24.3, 22.3) 0.932

For peer review only - http://bmjopen.bmj.com/site/about/guidelines.xhtml

Supplementary table 1: Recorded insight and clinical outcomes at 12, 24, 36, 48 and 60 months

	Recorded poor insight	
	Present (n=991)	Absent (n=1,035)
**12 months**		
Psychiatric hospitalisation (%)	22.6	28.6
Compulsory hospitalisation (%)	18.1	23.7
Number of unique antipsychotics (mean, SD)	1.39 (1.18)	1.77 (1.12)
Number of days spent as an inpatient (mean, SD)	19.7 (47.8)	29.3 (54.0)
**24 months**		
Psychiatric hospitalisation (%)	31.9	39.7
Compulsory hospitalisation (%)	25.7	31.2
Number of unique antipsychotics (mean, SD)	1.9 (1.45)	1.92 (1.44)
Number of days spent as an inpatient (mean, SD)	41.44 (89.1)	42.35 (89.8)
**36 months**		
Psychiatric hospitalisation (%)	37.8	43.3
Compulsory hospitalisation (%)	26.2	34.3
Number of unique antipsychotics (mean, SD)	2.07 (1.65)	2.13 (1.6)
Number of days spent as an inpatient (mean, SD)	56.7 (120.4)	58.9 (124.2)
**48 months**		
Psychiatric hospitalisation (%)	41.7	48.1
Compulsory hospitalisation (%)	33.7	39.1
Number of unique antipsychotics (mean, SD)	2.2 (1.78)	2.3 (1.70)

For peer review only

Number of days spent as an inpatient (mean, SD)	69.1 (148.7)	73.8 (158.5)
**60 months**		
Psychiatric hospitalisation (%)	44.2	51.2
Compulsory hospitalisation (%)	36.9	39.6
Number of unique antipsychotics (mean, SD)	2.3 (1.92)	2.4 (1.78)
Number of days spent as an inpatient (mean, SD)	80.37 (178.1)	86.1 (193.5)

For peer review only

STROBE Statement—checklist of items that should be included in reports of observational studies

	Item No	Recommendation
**Title and abstract**	1	(*a*) Indicate the study's design with a commonly used term in the title or the abstract **PAGE 1**
		(*b*) Provide in the abstract an informative and balanced summary of what was done and what was found **PAGE 2**
**Introduction**		
Background/rationale	2	Explain the scientific background and rationale for the investigation being reported **PAGE 4**
Objectives	3	State specific objectives, including any pre-specified hypotheses **PAGE 4**
**Methods**		
Study design	4	Present key elements of study design early in the paper **PAGE 5**
Setting	5	Describe the setting, locations, and relevant dates, including periods of recruitment, exposure, follow-up, and data collection **PAGES 5**
Participants	6	(*a*) *Cohort study*—Give the eligibility criteria, and the sources and methods of selection of participants. Describe methods of follow-up *Case-control study*—Give the eligibility criteria, and the sources and methods of case ascertainment and control selection. Give the rationale for the choice of cases and controls *Cross-sectional study*—Give the eligibility criteria, and the sources and methods of selection of participants
		(*b*) *Cohort study*—For matched studies, give matching criteria and number of exposed and unexposed *Case-control study*—For matched studies, give matching criteria and the number of controls per case **PAGE 6**
Variables	7	Clearly define all outcomes, exposures, predictors, potential confounders, and effect modifiers. Give diagnostic criteria, if applicable **PAGES 6 and 7**
Data sources/ measurement	8*	For each variable of interest, give sources of data and details of methods of assessment (measurement). Describe comparability of assessment methods if there is more than one group **PAGE 5**
Bias	9	Describe any efforts to address potential sources of bias **PAGE 7**
Study size	10	Explain how the study size was arrived at **PAGES 5 and 6**
Quantitative variables	11	Explain how quantitative variables were handled in the analyses. If applicable, describe which groupings were chosen and why **PAGES 6 and7**
Statistical methods	12	(*a*) Describe all statistical methods, including those used to control for confounding **PAGES 6 and 7**

For peer review only - http://bmjopen.bmj.com/site/about/guidelines.xhtml
For peer review only

		(*b*) Describe any methods used to examine subgroups and interactions<br>**PAGES 6 and 7**
		(*c*) Explain how missing data were addressed
		(*d*) *Cohort study*—If applicable, explain how loss to follow-up was addressed<br>*Case-control study*—If applicable, explain how matching of cases and controls was addressed<br>*Cross-sectional study*—If applicable, describe analytical methods taking account of sampling strategy
		(*e*) Describe any sensitivity analyses
**Results**		
Participants	13*	**PAGE 8**
Descriptive data	14*	**PAGE 8**
Outcome data	15*	**PAGE 8**
Main results	16	(*a*) Give unadjusted estimates and, if applicable, confounder-adjusted estimates and their precision (eg, 95% confidence interval). Make clear which confounders were adjusted for and why they were included<br>**PAGE 8**
		(*b*) Report category boundaries when continuous variables were categorized<br>**PAGE 8**
		(*c*) If relevant, consider translating estimates of relative risk into absolute risk for a meaningful time period
Other analyses	17	Report other analyses done—eg analyses of subgroups and interactions, and sensitivity analyses
**Discussion**		
Key results	18	Summarise key results with reference to study objectives<br>**PAGE 9**
Limitations	19	Discuss limitations of the study, taking into account sources of potential bias or imprecision. Discuss both direction and magnitude of any potential bias<br>**PAGES 9 and 10**
Interpretation	20	Give a cautious overall interpretation of results considering objectives, limitations, multiplicity of analyses, results from similar studies, and other relevant evidence<br>**PAGES 9 and 10**
Generalisability	21	Discuss the generalisability (external validity) of the study results<br>**PAGES 9 and 10**
**Other information**		
Funding	22	Give the source of funding and the role of the funders for the present study and, if applicable, for the original study on which the present article is based<br>**PAGE 12**

For peer review only

*Give information separately for cases and controls in case-control studies and, if applicable, for exposed and unexposed groups in cohort and cross-sectional studies.

Note: An Explanation and Elaboration article discusses each checklist item and gives methodological background and published examples of transparent reporting. The STROBE checklist is best used in conjunction with this article (freely available on the Web sites of PLoS Medicine at http://www.plosmedicine.org/, Annals of Internal Medicine at http://www.annals.org/, and Epidemiology at http://www.epidem.com/). Information on the STROBE Initiative is available at www.strobe-statement.org.

For peer review only

For peer review only - http://bmjopen.bmj.com/site/about/guidelines.xhtml
